# Preoperative hydronephrosis is an independent protective factor of renal function decline after nephroureterectomy for upper tract urothelial carcinoma

**DOI:** 10.3389/fonc.2023.944321

**Published:** 2023-02-24

**Authors:** Pai-Yu Cheng, Hsiang-Ying Lee, Wei-Ming Li, Steven K. Huang, Chien-Liang Liu, I-Hsuan Alan Chen, Jen-Tai Lin, Chi-Wen Lo, Chih-Chin Yu, Shian-Shiang Wang, Chuan-Shu Chen, Jen-Shu Tseng, Wun-Rong Lin, Jou Yeong-Chin, Ian-Seng Cheong, Yuan-Hong Jiang, Yu Khun Lee, Yung-Tai Chen, Shin-Hong Chen, Bing-Juin Chiang, Thomas Y. Hsueh, Chao-Yuan Huang, Chia-Chang Wu, Wei Yu Lin, Yao-Chou Tsai, Kai-Jie Yu, Chi-Ping Huang, Yi-You Huang, Chung-You Tsai

**Affiliations:** ^1^ Institute of Biomedical Engineering, National Taiwan University, Taipei, Taiwan; ^2^ Divisions of Urology, Department of Surgery, Far Eastern Memorial Hospital, New Taipei City, Taiwan; ^3^ Department of Urology, Kaohsiung Medical University Hospital, Kaohsiung, Taiwan; ^4^ Department of Urology, School of Medicine, College of Medicine, Kaohsiung Medical University, Kaohsiung, Taiwan; ^5^ Department of Urology, Kaohsiung Municipal Ta-Tung Hospital, Kaohsiung, Taiwan; ^6^ Graduate Institute of Clinical Medicine, College of Medicine, Kaohsiung Medical University, Kaohsiung, Taiwan; ^7^ Graduate Institute of Medicine, College of Medicine, Kaohsiung Medical University, Kaohsiung, Taiwan; ^8^ Department of Urology, Ministry of Health and Welfare Pingtung Hospital, Pingtung, Taiwan; ^9^ Cohort Research Center, Kaohsiung Medical University, Kaohsiung, Taiwan; ^10^ Division of Urology, Department of Surgery, Chi Mei Medical Center, Tainan, Taiwan; ^11^ Department of Medical Science Industries, College of Health Sciences, Chang Jung Christian University, Tainan, Taiwan; ^12^ Division of Urology, Department of Surgery, Kaohsiung Veterans General Hospital, Kaohsiung, Taiwan; ^13^ Division of Urology, Department of Surgery, Taipei Tzu Chi Hospital, The Buddhist Medical Foundation, New Taipei City, Taiwan; ^14^ School of Medicine, Buddhist Tzu Chi University, Hualien, Taiwan; ^15^ Division of Urology, Department of Surgery, Taichung Veterans General Hospital, Taichung, Taiwan; ^16^ Institute of Medicine, Chung Shan Medical University, Taichung, Taiwan; ^17^ Department of Applied Chemistry, National Chi Nan University, Nantou, Taiwan; ^18^ Department of Senior Citizen Service Management, National Taichung University of Science and Technology, Taichung, Taiwan; ^19^ Department of Urology, MacKay Memorial Hospital, Taipei, Taiwan; ^20^ Mackay Medical College, Taipei, Taiwan; ^21^ Institute of Biomedical Informatics, National Yang Ming Chiao Tung University, Taipei, Taiwan; ^22^ Department of Urology, Ditmanson Medical Foundation Chiayi Christian Hospital, Chiayi, Taiwan; ^23^ Department of Health and Nutrition Biotechnology, Asian University, Taichung, Taiwan; ^24^ Department of Urology, Hualien Tzu Chi Hospital, Buddhist Tzu Chi Medical Foundation and Tzu Chi University, Hualien, Taiwan; ^25^ Department of Urology Taiwan Adventist Hospital, Taipei, Taiwan; ^26^ College of Medicine, Fu-Jen Catholic University, New Taipei City, Taiwan; ^27^ Department of Urology, Cardinal Tien Hospital, New Taipei City, Taiwan; ^28^ Department of Life Science, College of Science, National Taiwan Normal University, Taipei, Taiwan; ^29^ Division of Urology, Department of Surgery, Taipei City Hospital renai branch, Taipei, Taiwan; ^30^ Department of Urology, School of Medicine, National Yang Ming Chiao Tung University, Taipei, Taiwan; ^31^ Department of Urology, College of Medicine, National Taiwan University Hospital, Taipei, Taiwan; ^32^ Department of Urology, Shuang Ho Hospital, Taipei Medical University, New Taipei City, Taiwan; ^33^ Department of Urology, School of Medicine, College of Medicine, Taipei Medical University, Taipei, Taiwan; ^34^ TMU Research Center of Urology and Kidney (TMU-RCUK), Taipei Medical University, Taipei, Taiwan; ^35^ Division of Urology, Department of Surgery, Chang Gung Memorial Hospital, Chia-Yi, Taiwan; ^36^ Chang Gung University of Science and Technology, Chia-Yi, Taiwan; ^37^ Department of Medicine, College of Medicine, Chang Gung University, Taoyuan, Taiwan; ^38^ Department of Surgery, Taipei Tzu chi Hospital, The Buddhist Tzu Chi Medical Foundation, New Taipei City, Taiwan; ^39^ Department of Urology, Taipei Medical University Hospital, Taipei Medical University, Taipei, Taiwan; ^40^ Division of Urology, Department of Surgery, Chang Gung Memorial Hospital at Linkou, Taoyuan, Taiwan; ^41^ School of Medicine, College of Medicine, Chang Gung University, Taoyuan, Taiwan; ^42^ Department of Chemical Engineering and Biotechnology and Graduate Institute of Biochemical and Biomedical Engineering, National Taipei University of Technology, Taipei, Taiwan; ^43^ Department of Urology, China Medical University and Hospital, Taichung, Taiwan; ^44^ School of Medicine, China Medical University, Taichung, Taiwan; ^45^ Department of Electrical Engineering, Yuan Ze University, Taoyuan, Taiwan

**Keywords:** hydronephrosis, nephroureterectomy, renal function, upper tract urothelial carcinoma, chronic kidney disease, adjuvant therapy, cisplatin-based chemotherapy, immune-oncology therapy

## Abstract

**Objectives:**

To evaluate the predictive role of pre-nephroureterectomy (NU) hydronephrosis on post-NU renal function (RF) change and preserved eligibility rate for adjuvant therapy in patients with upper tract urothelial carcinoma (UTUC).

**Patients and methods:**

This retrospective study collected data of 1018 patients from the Taiwan UTUC Collaboration Group registry of 26 institutions. The patients were divided into two groups based on the absence or presence of pre-NU hydronephrosis. Estimated glomerular filtration rate (eGFR) was calculated pre- and post-NU respectively. The one month post-NU RF change, chronic kidney disease (CKD) progression, and the preserved eligibility rate for adjuvant therapy were compared for each CKD stage.

**Results:**

404 (39.2%) patients without and 614 (60.8%) patients with pre-NU hydronephrosis were enrolled. The median post-NU change in the eGFR was significantly lower in the hydronephrosis group (-3.84 versus -12.88, p<0.001). Pre-NU hydronephrosis was associated with a lower post-NU CKD progression rate (33.1% versus 50.7%, *p*< 0.001) and was an independent protective factor for RF decline after covariate adjustment (OR=0.46, *p*<0.001). Patients with pre-NU hydronephrosis had a higher preserved eligibility rate for either adjuvant cisplatin-based chemotherapy (OR=3.09, 95%CI 1.95–4.69) or immune-oncology therapy (OR=2.31, 95%CI 1.23–4.34).

**Conclusion:**

Pre-NU hydronephrosis is an independent protective predictor for post-NU RF decline, CKD progression, and eligibility for adjuvant therapy. With cautious selection for those unfavorably prognostic, non-metastatic UTUC patients with preoperative hydronephrosis, adjuvant rather than neoadjuvant therapy could be considered due to higher chance of preserving eligibility.

## Introduction

Upper tract urothelial carcinoma (UTUC), involving renal pelvis or ureter instead of the bladder, accounts for 5-10% of all urothelial cancers (1). As such, UTUC is a relatively uncommon urothelial carcinoma. Its prevalence was reported to be 101 per million people in European countries (2). The current definitive treatment for localized UTUC is radical nephroureterectomy (NU) combined with bladder cuff excision. Nephron sparing surgery could only be reserved for patients with low risk UTUC as an alternative option, but not the standard of care (3). Since radical NU is inevitable for localized UTUC, post-NU renal function (RF) decline may be a major concern for patient prognosis and an obstacle to providing adjuvant therapy, such as chemotherapy or immuno-oncology (IO) therapy (4).

Radical nephrectomy has been well documented to have a negative impact on RF and may facilitate chronic kidney disease (CKD) development (5, 6). However, the current literature focusing on post-NU RF decline is relatively sparse. Instead of reporting RF quantitative measurements, it mainly focused on the RF eligibility for post NU adjuvant cisplatin-based chemotherapy (CBCT). Few studies have fully elucidated post-NU RF changes in patients with different CKD stages (I to V). Along with various emerging post-NU adjuvant therapy modalities addressing different RF constraints, a larger-scale study is required to further investigate the actual impact of NU on RF decline for patients with different pre-NU CKD stage.

For UTUC patients with pathological confirmation of muscularis invasion or regional lymph node involvement, adjuvant chemotherapy or IO therapy demonstrated benefit in prolonging disease free survival (7–9). However, the Galsky criteria defined an eligible RF for CBCT was eGFR ≥ 60 mL/min/1.73 m^2^, whereas eGFR ≥ 30 mL/min/1.73m^2^ was considered suitable for Nivolumab treatment since current evidences were very limited for patients with severe renal impairment (eGFR< 30 mL/min/1.73m2) (10, 11). Therefore, a remarkable decline of RF after surgery may preclude those patients from receiving adjuvant therapy. Thus, we aimed to compare the eligibility rates between neoadjuvant and adjuvant therapies based on the different RF criteria.

Ipsilateral pre-NU hydronephrosis has been proposed as an independent predictor of post-NU RF decline (12, 13). However, the predictive role of hydronephrosis for post-NU RF decline has not been fully explored in patients with different pre-NU CKD stages, especially stage III and IV. The association between pre-NU hydronephrosis and RF preservation for adjuvant treatment is of special interest to the community in order to make personalized treatment decisions. We hypothesized that pre-NU hydronephrosis was an independent protective factor for post-NU RF decline no matter which pre-NU CKD stage the patient was in. Hence, we aimed to identify the predictive role of pre-NU hydronephrosis in each CKD stratum by comprehensively analyzing a multi-institutional registry database. This would assist in decision-making when considering neoadjuvant or adjuvant therapy in combination with NU for non-metastatic UTUC patients with unfavorable prognosis.

## Patients and methods

### Database information

A multicenter registry database, created by the Taiwan UTUC Collaboration Group, was used to analyze patients with UTUC from 2001 to 2021 among 26 secondary and tertiary referral hospitals in Taiwan. The Internal Review Board of each participating hospital granted ethical approval and waived informed consent from all patients in the database. To ensure data consistency and accuracy and avoid discrepancies, consensus meetings were held and data acquisition protocols were established. According to the consensus meetings, 124 clinicopathological parameters were recorded. All identifiable personal information was removed to protect the privacy.

### Data collection

From March 01, 2001 to March 31, 2021, a total of 4016 patients with UTUC who undergo NU as the primary surgery were enrolled from the Taiwan UTUC Collaboration Group registry database for analysis. Patients with missing data on any of the variables for analysis were excluded (n = 2787). Patients younger than 18 years (n = 5), with end-stage renal disease prior to surgery (n = 156), who received kidney transplantation (n = 16), or had undergone NU previously (n = 10) were excluded. Patients who had received neoadjuvant chemotherapy (NAC) prior to surgery (n = 23) or who underwent bilateral NU simultaneously (n = 1) were also excluded from the study (**Supplementary Figure S1**).

Demographic data, including age, sex, comorbidities (hypertension, diabetes mellitus, or coronary artery disease), tumor site (right or left), location (renal pelvis only or ureter involvement), ipsilateral hydronephrosis (presence or absence), tumor focality (single or multiple), and tumor size, were collected. Ipsilateral hydronephrosis was defined as the hydronephrosis presenting on the same side as tumor involvement. Pathological tumor staging was recorded according to the eighth edition of the American Joint Committee on Cancer Staging Manual. Post-NU events such as sepsis or shock were also recorded (**Table 1**).

**Table 1:** Patient demographics grouped by pre-NU hydronephrosis.

Characteristics	Entire cohort n (%)	Pre-NU hydronephrosis		
		Absent (group 1)	Present (group 2)	*p* value
**Patients**	1018	404 (39.2)	614 (60.8)	
**Age, median (IQR)**	69 (62-76)	69 (61-75)	70 (62-76)	0.079
**Sex**				0.652
Male	480 (47.2)	194 (48.0)	286 (46.6)	
Female	538 (52.8)	210 (52.0)	328 (53.4)	
**Medical history**				
Hypertension	533 (52.4)	178 (44.1)	355 (57.8)	< 0.001^*^
Diabetes mellitus	317 (31.1)	127 (31.4)	190 (30.9)	0.869
Coronary artery disease	89 (8.7)	33 (8.2)	56 (9.1)	0.599
**Pre-NU renal function**				
eGFR, **mean (SD)**	54.7 (24.4)	57.0 (23.7)	53.2 (24.8)	0.016
**Pre-NU CKD stage**				0.071
I (eGFR ≥ 90)	100 (9.8)	41 (10.1)	59 (9.6)	
II (60 ≤ eGFR < 90)	316 (31.0)	139 (34.4)	177 (28.8)	
III (30 ≤ eGFR < 60)	431 (42.3)	171 (42.3)	260 (42.3)	
IV (15 ≤ eGFR < 30)	111 (10.9)	37 (9.2)	74 (12.1)	
V (eGFR < 15)	60 (5.9)	16 (4.0)	44 (7.2)	
Clinical presentation				
**Site**				0.457
Right	506 (49.7)	195 (48.3)	311 (50.7)	
Left	512 (50.3)	209 (51.7)	303 (49.3)	
**Location**				< 0.001^*^
Renal pelvis only	489 (48.0)	288 (71.3)	201 (32.7)	
Ureter involvement	529 (52.0)	116 (28.7)	413 (67.3)	
**Tumor size**				0.829
< 2cm	243 (23.9)	95 (23.5)	148 (24.1)	
≥ 2cm	775 (76.1)	309 (76.5)	466 (75.9)	
**Tumor focality**				0.061
Single	650 (63.9)	272 (67.3)	378 (61.6)	
Multiple	368 (36.1)	132 (32.7)	236 (38.4)	
**Pathologic T stage**				0.527
< pT3	547 (53.7)	222 (55.0)	325 (52.9)	
≥ pT3	471 (46.3)	182 (45.0)	289 (47.1)	
**Post-NU sepsis**	2 (0.2)	0 (0)	2 (0.3)	0.251
**Post-NU shock**	11 (1.1)	3 (0.7)	8 (1.3)	0.398

NU, Nephroureterectomy; CKD, Chronic kidney disease; IQR, Interquartile range.

*Statistically significant in χ2 test.

### RF measurements and outcome

For RF evaluation, eGFR was calculated using the CKD Epidemiology Collaboration (CKD-EPI) equation 2021, which incorporates serum creatinine, age, and sex for estimation (14).

eGFR (mL/min/1.73m^2^) = 142×Min (standardized Scrκ,1)α×Max (standardized Scrκ,1)−1.200×0.9938Age×1.012 [if female] ,

where Scr is serum creatinine, κ is 0.7 for women and 0.9 for men, and α is -0.241 for women and -0.302 for men. Min indicates the minimum SCr/κ or 1, and Max indicates the maximum SCr/κ or 1.

Three other equations for eGFR estimation, namely the CKD-EPI 2009, isotope-dilution mass spectrometry-modification of diet in renal disease (IDMS-MDRD), and Taiwan MDRD were also included to test the robustness of the statistical results (15–17).

The quantitative RF outcome, post-NU eGFR change (ΔeGFR), was calculated as

ΔeGFR = (eGFR within one month post-NU) – (pre-NU eGFR).

A more negative ΔeGFR indicated an increased RF decline post-NU. Pre-NU serum creatinine levels were measured upon admission for surgery. Post-NU serum creatinine levels were measured during clinic follow-up within one month after surgery.

The post-NU CKD progression was a binary outcome variable and defined as the post-NU eGFR decline either greater than or equal to 25% from the baseline (18, 19). Furthermore, the eGFR was stratified into CKD stages I to V according to the Kidney Disease: Improving Global Outcomes 2012 clinical practice guidelines (5). For testing the robustness of our findings, another definition of CKD progression was also adopted and was defined as the post-NU incremental progression to higher CKD stage.

Although an eGFR greater than or equal to 60 mL/min/1.73m^2^ was considered eligible for CBCT, a more lenient criterion of eGFR greater than or equal to 50 mL/min/1.73m^2^ was also adopted (10, 20). On the basis of the RF criterion, patients with UTUC who were judged to be eligible for neoadjuvant treatment preoperatively but underwent NU first may turn ineligible for adjuvant treatment due to post-NU RF decline. Two indicators, preserved and overall eligibility rates

Preserved eligibility rate =


$$\frac{\text{Number of patients eligible for adjuvant treatment post}-\text{NU}}{\text{Number of patients eligible for neoadjuvant treatment pre}-\text{NU}}$$

Overall eligibility rate = (Number of eligible patients)/(Number of cohorts), were applied for the analysis.

### Statistical analysis

All categorical demographic variables were compared using χ2 tests. Numerical variables without a normal distribution are reported as median and interquartile range (IQR) and compared using the Mann–Whitney U test. Continuous outcome variables with normal distribution are reported as means and standard deviations and compared using Student’s t-test. Univariable and multivariable linear regression analyses were performed for the continuous outcome variable, ΔeGFR, and univariable and multivariable logistic regression analyses were performed for the binary outcome variable, CKD progression. All p-values were two-tailed, and *p*< 0.05 was considered statistically significant at 95% confidence intervals (CI). SPSS version 25 and R version 4.0.3 software were used for the statistical analysis. GraphPad Prism 9 software was used to generate graphs and plots.

## Results

### Clinicopathological characteristics of the study cohort and patient groups

After implementing the exclusion criteria described above, 1018 patients with UTUC were enrolled in the study cohort. The median age was 69.0 years (IQR 62–76 years), and 47.2% of the patients were male. The patients were divided into two groups based on the absence or presence of pre-NU hydronephrosis. Group 1 consisted of 404 (39.2%) patients without pre-NU hydronephrosis and group 2 consisted of 614 (60.8%) patients with pre-NU hydronephrosis. The number of patients with CKD stages I-V pre-NU were 100 (9.8%), 316 (31.0%), 431 (42.3%), 111 (10.9%), and 60 (5.9%), respectively. Comparing the clinicopathologic characteristics between the two groups, most characteristics did not show any statistical difference, except for hypertension (*p*< 0.001) and UTUC involving the ureter (*p*< 0.001) (**Table 1**).

The median pre-NU eGFR was 54.02 mL/min/1.73m^2^ (IQR 37.75–71.36 mL/min/1.73m^2^), and the median post-NU eGFR was 44.33 mL/min/1.73m^2^ (IQR 29.22–58.48 mL/min/1.73m^2^). The mean ΔeGFR of the entire cohort within one month post-NU was -10.14 mL/min/1.73m^2^ (range -79.80 to 41.00 mL/min/1.73m^2^), which represented a marked postoperative eGFR decline.

### Distribution and comparison of post-NU ΔeGFR between the patient groups

**Figure 1:**
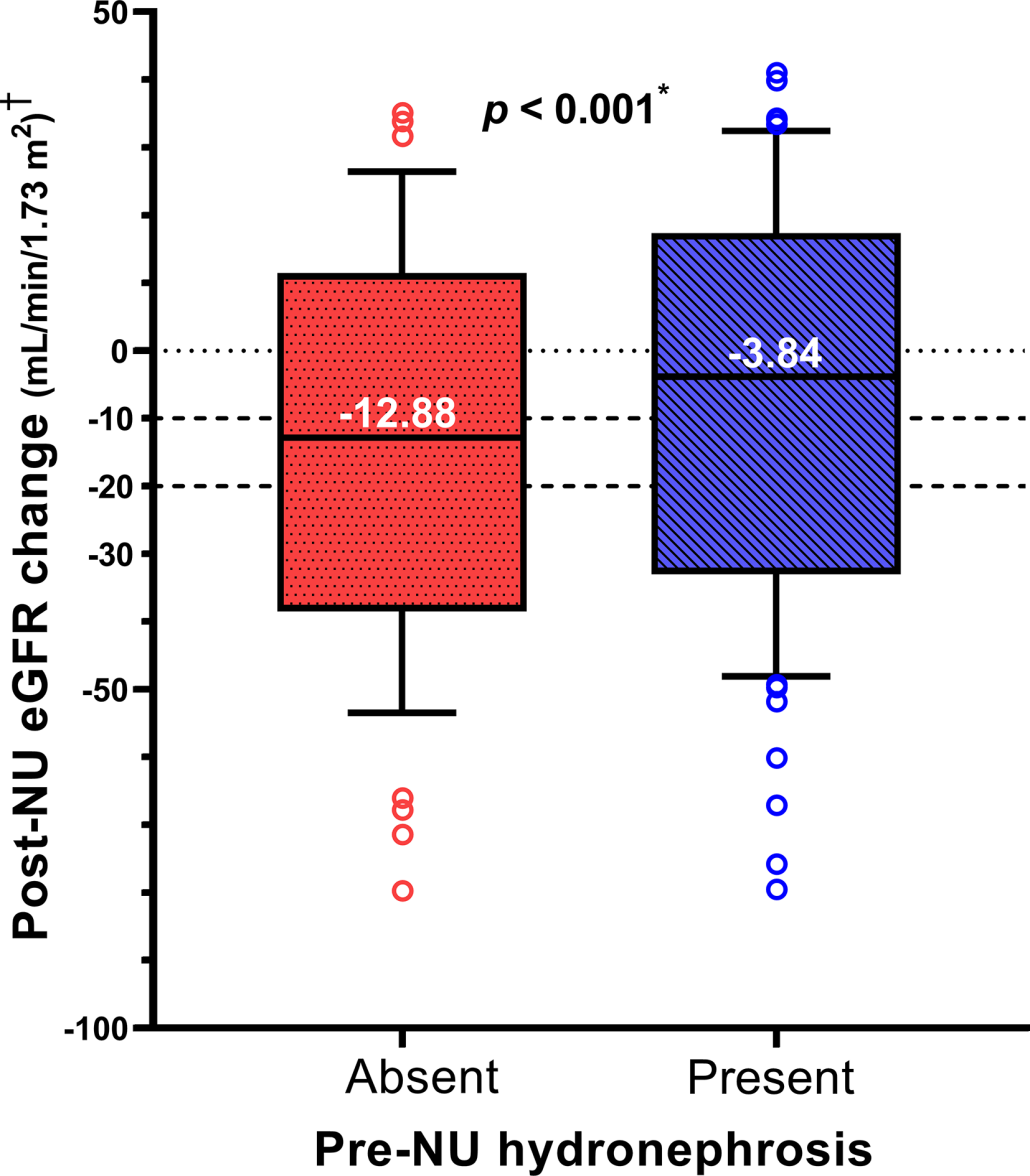
The box plot compares post-NU ΔeGFR between the two patient groups within one month post-NU. † Post-NU eGFR change = ΔeGFR = (1 month post-NU eGFR) – (pre-NU eGFR). *Statistically significant using Mann-Whitney U test.

The distribution of post-NU ΔeGFR was compared between the two patient groups. The median ΔeGFR values in the absence and presence of pre-NU hydronephrosis were -12.88 and -3.84 mL/min/1.73m^2^, respectively. Patients with pre-NU hydronephrosis (group2) had a significantly lower (*p*< 0.001) eGFR decline post-NU than those without pre-NU hydronephrosis (group1), as determined by the Mann–Whitney U test (**Figure 1**).

Linear regression was performed to quantify the impact of hydronephrosis on ΔeGFR. In univariable analysis, pre-NU hydronephrosis was significantly associated with a lower eGFR decline post-NU (Coefficient B value = +6.06, 95% CI 4.05–8.07). Multivariable linear regression analysis was performed to adjust for other covariates that may affect ΔeGFR. When comparing patients with and without hydronephrosis, pre-NU hydronephrosis positively correlated with post-NU ΔeGFR even after adjustment (**Table 2**; B value = +5.11, 95% CI 3.42–6.81). The positive coefficient B of ΔeGFR indicated a protective effect of pre-NU hydronephrosis. Additionally, hydronephrosis had the strongest effect (standardized beta = 0.15) on post-NU ΔeGFR among all the analyzed covariates.

**Table 2:** Multiple linear regression of post-NU eGFR change^†^ (n = 1018).

Variables^¶^	Unstandardized Coefficients		Standardized Coefficients				Collinearity Statistics	
	B	Standard error	β	T	*p* value	95% CI	Tolerance	VIF
**Constant**	19.95	3.26		6.12	<0.001^*^	13.55 – 26.35		
**Hydronephrosis** ^†^	5.11	0.87	0.15	5.91	<0.001^*^	3.42 – 6.81	0.98	1.03
**Age**	-0.21	0.04	-0.14	-5.00	<0.001^*^	-0.29 – -0.13	0.88	1.13
**Hypertension**	-2.43	0.87	-0.08	-2.80	0.005^*^	-4.13 – -0.72	0.93	1.08
**Multifocality**	1.80	0.89	0.05	2.04	0.042^*^	0.07 – 3.54	0.96	1.04
**Pathologic T stage ≥ pT3**	3.03	0.85	0.09	3.56	<0.001^*^	1.36 – 4.70	0.97	1.03
**Pre-NU eGFR**	-0.37	0.02	-0.55	-20.37	<0.001^*^	-0.40 – -0.33	0.91	1.10

Adjusted R^2^ = 0.328, p< 0.001.

CI,Confidence interval; VIF, Variance inflation factor.

†Post-NU eGFR change =ΔeGFR = (1 month post-NU eGFR) – (pre-NU eGFR).

¶Dichotomous variable compared to the opposite character, eg: hydronephrosis vs no hydronephrosis (reference).

*Statistically significant.

To test the robustness of the post-NU ΔeGFR comparison findings, four different eGFR estimation equations were applied: CKD-EPI 2021, CKD-EPI 2009, IDMS-MDRD, and the Taiwan MDRD. The above statistical significance was consistently valid in each case.

The scatter plot of post-NU ΔeGFR versus pre-NU eGFR demonstrates the distributions of the patients in the two groups (**Supplementary Figure S2**). The negative slope of the regression lines indicates that the better the patients’ RF pre-NU, the more would be their eGFR decline post-NU. Furthermore, the eGFR slope decline in patients with pre-NU hydronephrosis was lesser compared to those without (-0.303 vs -0.398, *p* = 0.009), which indicates that the presence of hydronephrosis played a protective role against RF decline.

### Association between CKD progression at one month post-NU and the presence of pre-NU hydronephrosis

**Figure 2:**
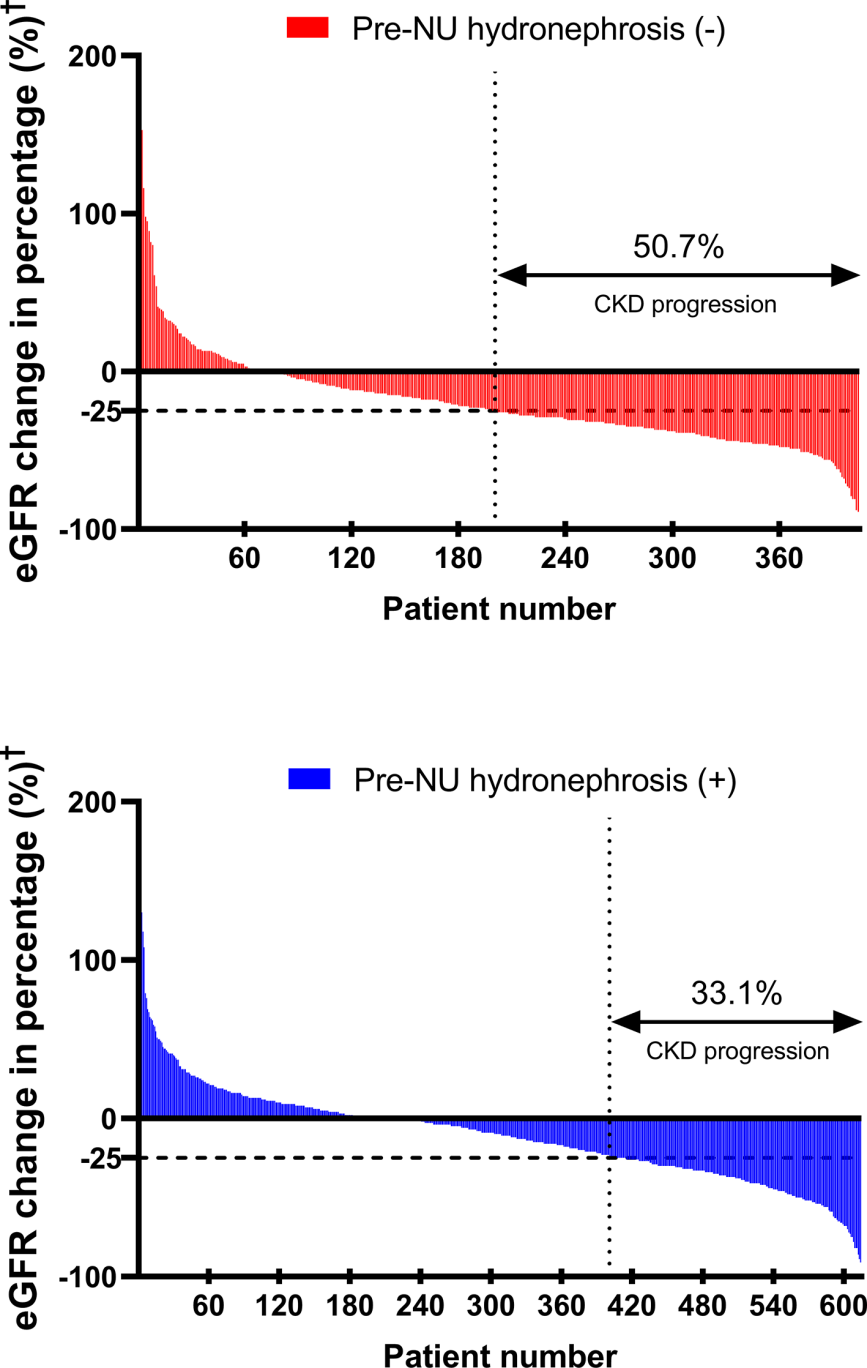
The waterfall plots compare CKD progression^‡^ rates between the two patient groups. †Post-NU eGFR change (%) = $\frac{\left(1\;month\;post-NU\;eGFR\right)\;–\;\left(pre-NU\;eGFR\right)}{\left(pre-NU\;eGFR\right)}$ . ‡CKD progression = post-NU eGFR decline ≥ 25% from baseline.

Post-NU CKD progression was defined as eGFR decline greater than or equal to 25% at one month post-NU. Pre-NU hydronephrosis was associated with a lower CKD progression rate post-NU (**Figure 2**; 33.1% in group 2 versus 50.7% in group 1, χ^2^ test, *p*< 0.001).

Pre-NU hydronephrosis was associated with less CKD progression according to the univariable analysis [**Table 3**; odds ratio (OR) = 0.48, 95% CI 0.37–0.62, *p*< 0.001]. After adjustment for other covariates in the multivariable analysis, hydronephrosis was a strong independent protective factor for CKD progression (**Table 3**; OR = 0.46, 95% CI 0.35–0.60, *p*< 0.001). The association was also confirmed either in univariable (OR = 0.51, 95% CI 0.39–0.66, *p*< 0.001) and multivariable analysis (OR = 0.50, 95% CI 0.39–0.65, *p*< 0.001) when redefining the CKD progression as the post-NU incremental progression to higher CKD stage (**Supplementary Table S1**).

**Table 3:** Logistic regression of post-NU CKD progression^†^ (↓eGFR ≥25%) (n = 1018).

Variable^¶^	Univariable analysis			Multivariable analysis		
	OR	(95% CI)	*p* value	OR	(95% CI)	*p* value
**Hydronephrosis**	0.48	(0.37 – 0.62)	<0.001*	0.46	(0.35 – 0.60)	<0.001*
**Age**	1.01	(1.00 – 1.02)	0.087	–	–	–
**Women**	1.08	(0.84 – 1.38)	0.575	–	–	–
**Hypertension**	1.27	(0.99 – 1.63)	0.066	1.34	(1.03 – 1.75)	0.032*
**Diabetes**	1.38	(1.05 – 1.80)	0.019*	–	–	–
**Coronary artery disease**	1.60	(1.03 – 2.47)	0.036*	1.57	(1.00 – 2.48)	0.054
**Multifocality**	0.68	(0.52 – 0.89)	0.004*	0.77	(0.58 – 1.01)	0.063
**Pathologic T stage ≥ pT3**	0.56	(0.43 – 0.72)	<0.001*	0.46	(0.35 – 0.60)	<0.001*
**Post-NU shock**	1.25	(0.38 – 4.12)	0.715	–	–	–

OR: Odds ratio.

†CKD progression = post-NU eGFR decline ≥ 25% from baseline.

^¶^Dichotomous variable compared to the opposite character, eg: hydronephrosis vs no hydronephrosis (reference).

*Statistically significant.

### Comparison of post-NU ΔeGFR between the two groups stratified by pre-NU CKD stage

**Figure 3:**
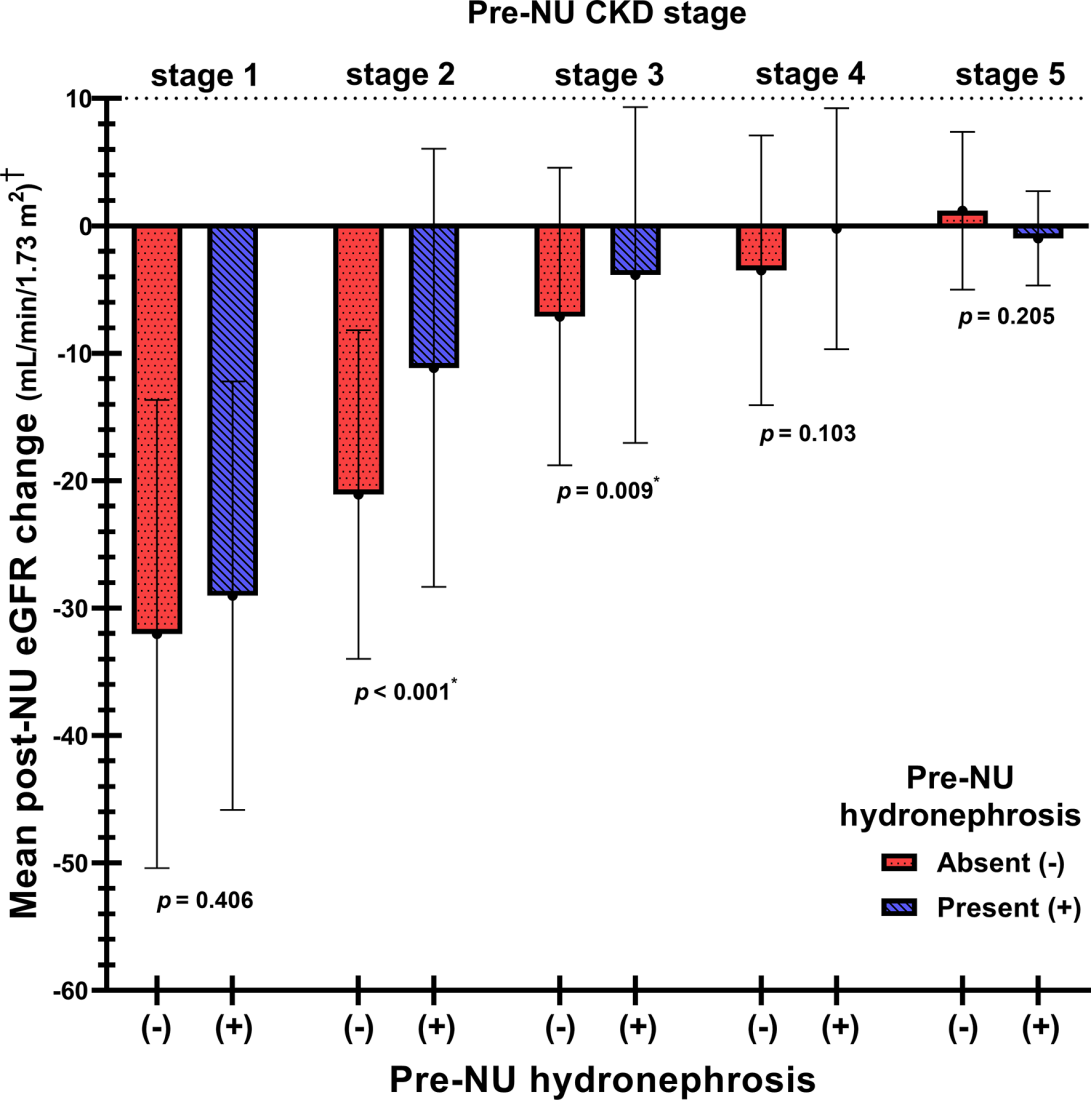
The comparison of mean post-NU ΔeGFR between the two patient groups stratified by pre-NU CKD stages. †Post-NU ΔeGFR = (1 month post-NU eGFR) – (pre-NU eGFR). *Statistically significant using Student’s t-test.

ΔeGFR was further analyzed and stratified into stages I to V CKD based on pre-NU eGFR. The mean ΔeGFR values were -30.26 (-31.1%), -15.52 (-21.0%), -5.15 (-11.0%), -1.31 (-6.3%), -0.41 (4.0%) in patients with stages I to V CKD, respectively (**Supplementary Table S2**). The comparison of mean post-NU ΔeGFR between the two groups for each CKD stage revealed that pre-NU hydronephrosis patients with stages II (*p<* 0.001) and III (*p* = 0.009) CKD had significantly lower eGFR decline post-NU compared to the pre-NU patients without hydronephrosis. However, significant differences were not observed between patients of the two groups with stage I, IV and V CKD (**Figure 3**).

While adjusting for age, hypertension, tumor focality, pathological p stage and pre-NU eGFR by multivariable linear regression, the pre-NU hydronephrosis patients with stages II and III CKD demonstrated significantly less eGFR decline post-NU, indicated by the coefficient B-values 9.27 (*p<* 0.001) and 3.79 (*p* = 0.002), respectively (**Supplementary Table S2**). Additionally, the significantly positive coefficient B for the ΔeGFR indicated the protective effect of pre-NU hydronephrosis in patients with stages II and III CKD. Furthermore, a protective effect with marginal trend towards significance (*p* = 0.054) was observed in pre-NU hydronephrosis patients with stage IV CKD.

### Eligibility for post-NU CBCT based on RF and the positive effect of pre-NU hydronephrosis

**Figure 4:**
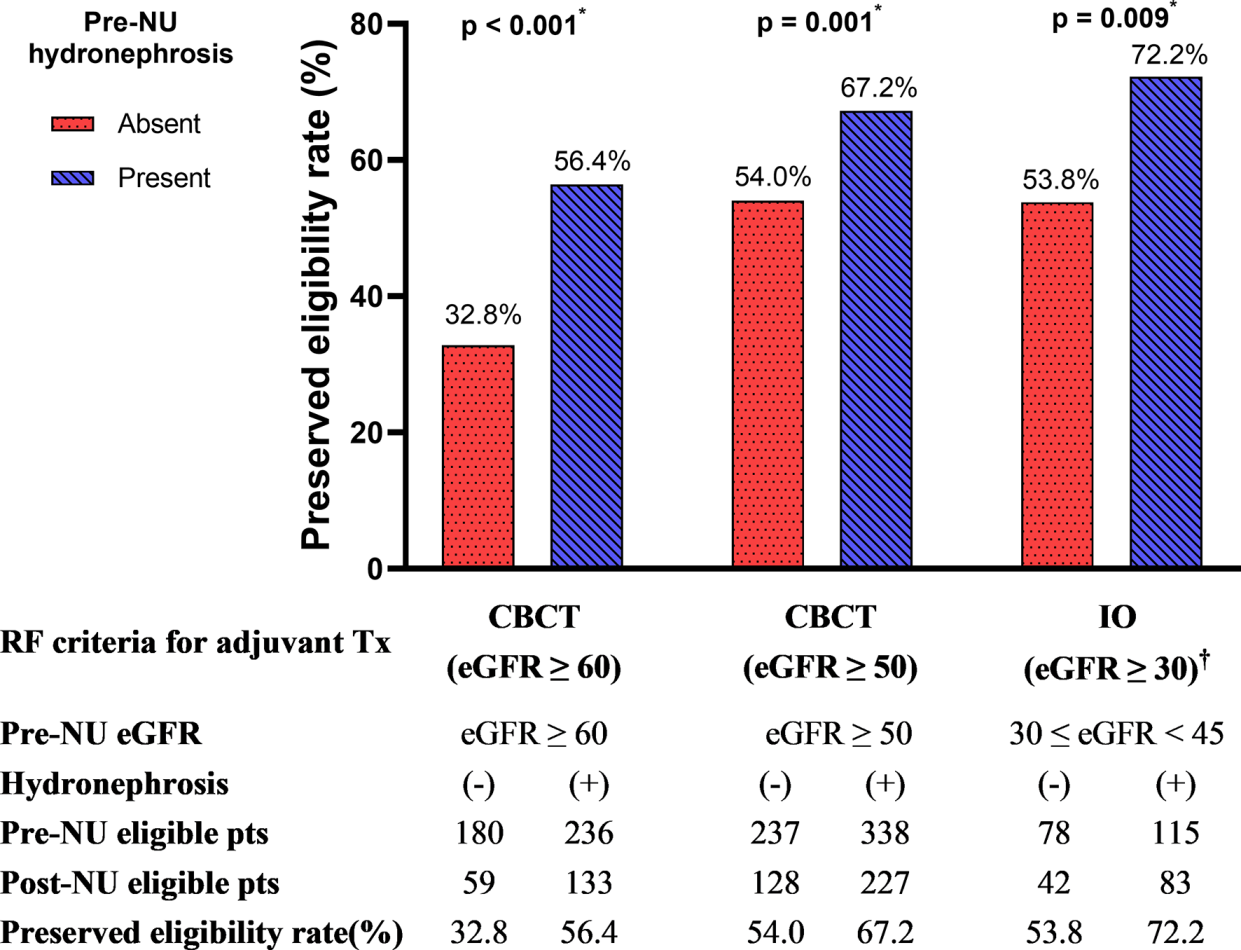
The preserved eligibility of adjuvant treatment for post-NU patients grouped by the absence and presence of pre-NU hydronephrosis. CBCT, cisplatin-based chemotherapy; IO, immuno-oncology therapy; RF, renal function. † No evidence-supported eligibility for IO therapy if eGFR< 30 mL/min/1.73m^2.^ * Statistically significant using χ2 test.

According to the RF criteria, 416 patients in the cohort were judged to be eligible for CBCT (eGFR ≥ 60 mL/min/1.73m^2^) pre-NU. However, only 192 (46.2%) patients preserved their eligibility for adjuvant CBCT one month post-NU. The preserved eligibility rate of patients without and with hydronephrosis were 32.8% and 56.4%, respectively (**Figure 4**). Therefore, patients with pre-NU hydronephrosis had a significantly higher preserved eligibility rate postoperatively for adjuvant CBCT (χ² tests, *p*< 0.001). Moreover, hydronephrosis was a significant protective predictor of preserved eligibility for adjuvant CBCT according to both univariable (OR = 2.65, 95% CI 1.77-3.97, *p*< 0.001) and multivariable (OR = 3.09, 95% CI 1.95–4.69, *p*< 0.001) analyses (**Table 4**). Conversely, age ≥ 70 years was a significant risk factor for retaining adjuvant CBCT eligibility (OR = 0.46, 95% CI 0.29-0.72, *p* = 0.001).

**Table 4:** Logistic regression of preserved RF eligibility for post-NU adjuvant therapy.

Variables^¶^	Univariable			Multivariable		
	OR	(95% CI)	*p* value	OR	(95% CI)	*p* value
RF eligible for adjuvant CBCT (eGFR ≥ 60) (192/416)						
**Hydronephrosis**	2.65	(1.77 – 3.97)	<0.001*	3.09	(1.95 – 4.69)	<0.001*
**Age ≥ 70**	0.41	(0.27 – 0.62)	<0.001*	0.46	(0.29 – 0.72)	0.001*
**Diabetes**	0.47	(0.29 – 0.75)	0.002*	0.50	(0.30 – 0.84)	0.009*
**Pre-NU eGFR**	1.04	(1.03 – 1.06)	<0.001*	1.05	(1.03 – 1.06)	<0.001*
RF eligible for adjuvant CBCT (eGFR ≥ 50) (355/575)						
**Hydronephrosis**	1.74	(1.24 – 2.45)	0.001*	2.28	(1.56 – 3.35)	<0.001*
**Age ≥ 70**	0.43	(0.31 – 0.61)	<0.001*	0.54	(0.37 – 0.79)	0.001*
**Hypertension**	0.65	(0.46 – 0.91)	0.012*	0.64	(0.44 – 0.94)	0.022*
**Pre-NU eGFR**	1.05	(1.04 –1.07)	<0.001*	1.06	(1.04 – 1.07)	<0.001*
RF eligible for adjuvant IO therapy (eGFR ≥ 30)^†^ (125/193)						
**Hydronephrosis**	2.22	(1.22 – 4.07)	0.009*	2.31	(1.23 – 4.34)	0.009*
**Pre-NU eGFR**	1.14	(1.06 – 1.22)	<0.001*	1.14	(1.06 – 1.23)	<0.001*

RF, renal function; CBCT, cisplatin-based chemotherapy; IO, immuno-oncology therapy.

¶Dichotomous variable compared to the opposite character, eg: hydronephrosis vs no hydronephrosis (reference).

**†**No solid evidence for eligibility of IO therapy if eGFR< 30 ml/min/1.73 m^2^.

*Statistically significant.

The overall eligibility rate for adjuvant CBCT was 40.9% pre-NU but decreased to 18.9% post-NU for the entire cohort. In addition, it was 44.6% pre-NU but decreased to 14.6% post-NU for patients without hydronephrosis, whereas 38.4% pre-NU decreased to 21.7% post-NU for patients with hydronephrosis.

We further compared the eligibility rates by varying the RF criterion. If the criterion for adjuvant CBCT eligibility was loosened to eGFR ≥ 50 mL/min/1.73m^2^, we found that 355 out of 575 (61.7%) patients were eligible for adjuvant CBCT. The preserved eligibility rates for patients without and with hydronephrosis were 54.0% and 67.2%, respectively (**Figure 4**; χ² test, *p* = 0.001). Pre-NU hydronephrosis was still a significant protective predictor of preserved eligibility for adjuvant CBCT postoperatively according to the multivariable analysis (**Table 4**; OR=2.28, 95% CI 1.56–3.35, *p*< 0.001).

### Evidence-supported eligibility for post-NU IO therapy based on RF and the positive effect of pre-NU hydronephrosis

Post-NU adjuvant IO therapy such as nivolumab may be considered for patients with UTUC who are ineligible for CBCT (8). However, current evidences were very limited for patients with severe renal impairment (eGFR< 30 mL/min/1.73m^2^) (11). Hence, evidence-supported RF criterion for adjuvant IO therapy was defined as eGFR ≥ 30 mL/min/1.73m^2^. Among the preoperative stage IIIa (30 mL/min/1.73m^2^ ≤ eGFR< 45 mL/min/1.73m^2^) CKD patients (n = 193, 19.0%) in our study cohort, 78 patients were without pre-NU hydronephrosis and 115 had pre-NU hydronephrosis. The preserved adjuvant IO therapy eligibility rates of patients without and with hydronephrosis were 53.8% and 72.2%, respectively (**Figure 4**; χ² test, *p* = 0.009). The presence of hydronephrosis was significantly associated with a greater chance of preserved eligibility for adjuvant IO therapy according to both univariable (OR = 2.22, 95% CI 1.22–4.07, *p* = 0.009) and multivariable logistic regression (OR = 2.31, 95% CI 1.23–4.34, *p* = 0.009) analysis (**Table 4**).

## Discussion

This study demonstrates that patients with pre-existing ipsilateral hydronephrosis who underwent NU for UTUC experienced less RF decline than those without hydronephrosis. We identified pre-NU hydronephrosis as an independent protective predictor for RF decline in patients with UTUC who received NU as the primary treatment, first, the positive coefficient B (5.11) in multivariable linear regression analysis for post-NU ΔeGFR; second, the low odds ratio (0.46) for CKD progression in the entire cohort; third, the protective effect in patients with pre-NU CKD stages II and III; and fourth, the higher preserved eligibility rate for adjuvant CBCT (OR = 3.09) or IO therapy (OR = 2.31).

Studies have shown that pre-NU hydronephrosis is an independent predictor for patients who are eligible for CBCT (12, 13). However, the number of patients in these studies was relatively small and mainly focused on their eligibility for adjuvant CBCT. Our research furthered the investigation of post-NU RF change not only in terms of a dichotomous eligibility outcome, but also in the quantitative measurement of ΔeGFR with effect size detection for each CKD stratum (stages I–V), thoroughly delineating the RF decline pattern in patients with UTUC who underwent NU. To our knowledge, this study comprised one of the largest multi-institutional patient cohorts focusing on post-NU RF decline. Additionally, this is the first study reporting the protective effect of hydronephrosis for post-NU RF decline in patients with stage III CKD.

This study aims to identify the clinically useful predictors for post-NU RF decline, which are available in daily pre-NU assessment. The association between ipsilateral pre-NU hydronephrosis and post-NU eGFR change was confirmed in the present study. However, the relationship was suggested to be association rather than causation. One possible mechanism by which hydronephrosis protects RF from decline could be explained as follows. Preoperative ipsilateral hydronephrosis represents obstruction of the urinary tract, which is usually caused by intramural growth of the tumor. Theoretically, the slowly developing urinary tract obstruction leads to ipsilateral kidney injury, and the contralateral kidney gradually compensates for the glomerular filtration. The compensation mechanism had been observed in animal models but no direct evidence had been proven in the human UTUC setting yet (21, 22). Therefore, further prospective study design with split-renal function measurement, such as Tc-99m DTPA renal scan, may be suggested to verify this hypothesis in the future.

Owing to the rare nature of the cancer, the prevalence of CKD in patients with UTUC, both pre- and post-NU, has been poorly reported. We have summarized the preoperative CKD stage distribution in **Table 1** and found that 42.3% of patients had stage III CKD and 59.1% had renal insufficiency (eGFR< 60 mL/min/1.73m^2^). Hence, CKD is highly prevalent in patients with UTUC. Only less than a half of patients (40.9%) had an adequate eGFR to receive neoadjuvant CBCT. These findings were consistent with those reported by Lane et al. (4) Furthermore, the overall eligibility rate of UTUC patients for adjuvant CBCT reduced to 18.9% post-NU. This result is similar to that reported from previous studies (4, 23).

Deterioration of RF post-NU is a major concern. Patients with UTUC have poor baseline RF and overall health condition than those with renal cell carcinoma (RCC) (24). This study revealed that the mean of ΔeGFR one month after NU were -30.26(-31.1%), -15.52(-21.0%), -5.15(-11.0%), -1.31(-6.3%) in CKD stage I to IV patients, respectively. In addition, the amplitude of RF decline was even greater in patients with UTUC without hydronephrosis. Although the long-term RF change was not reported due to data constraints, the short-term RF decline in patients with UTUC was an impediment and crucial to adjuvant therapy in the post-NU golden period (~1-3 months) (25). Thus, our findings confirmed the marked short-term RF decline post-NU, potentially eliminating the chance for adjuvant therapy (23). The long-term RF change post-NU has been inconsistently reported. Kaag et al. reported that the RF decline post-NU did not recover over time (26). However, in case of patients with RCC, a significant RF drop was also observed in the first 3 months after radical nephrectomy, which gradually recovered in the following 24-60 months after surgery (27, 28). However, it is doubtful to apply the findings in patients with RCC status post radical nephrectomy to patients with UTUC who had undergone NU.

The choice of neoadjuvant chemotherapy (NAC) or adjuvant chemotherapy (AC) in combination with definitive NU is still under debate (7). NAC offers favorable pathologic response, better tumor downstaging rate, and improves overall survival (OS) and cancer-specific survival (CSS) compared to NU alone (29). Similarly, utilization of AC yields longer OS, CSS, and even disease-free survival (DFS), compared to NU alone (9, 25, 29, 30). The results from the peri-operative chemotherapy versus surveillance in upper tract urothelial cancer (POUT) trial illustrated that gemcitabine-platinum combination chemotherapy within 90 days post-NU improves DFS and should be considered as a new standard of care in patients with locally advanced UTUC (9). A systematic review with meta-analysis supports the benefit of perioperative chemotherapy, and advocates AC more than NAC (31). Despite the level of evidence being stronger in favor of AC, NAC is still advocated because of the fact that less than 40% of the patients who were eligible for CBCT pre-NU remained eligible post-NU (7). However, predicting whether patients have higher tumor stage or adverse histologic characteristics pre-NU is arduous (32). The biopsies taken from ureteroscopy or retrograde intrarenal surgery tend to underestimate the actual disease severity (33). Additionally, for patients with non-muscle invasive UTUC, NAC might be an overtreatment. Finally, NAC may delay patients from receiving definitive surgical treatment, especially in those with chemo-resistant disease (34). Some patients may even become poor candidates for definitive NU after NAC.

Although AC is advocated more than NAC, the major limitation of AC delivery in UTUC is impaired RF post-NU. According to the Galsky criteria, reduced RF (eGFR< 60 mL/min/1.73m^2^) disqualified patients for CBCT (10, 31). However, our findings suggest that pre-NU hydronephrosis is an independent protective predictor from short-term RF decline. On the other hand, previous studies indicated that pre-NU hydronephrosis was associated with poor prognosis, such as disease progression, shortened OS, CSS, and DFS; thus, radical NU would be the prioritized choice in those patients (35–37). Delayed NU was also shown to be associated with a higher risk of disease recurrence and cancer-specific mortality in patients with ureteral urothelial carcinoma, the main cause of hydronephrosis (38). The present study revealed that 56.4% of patients with pre-NU hydronephrosis preserved eligibility for post-NU adjuvant CBCT, compared to 32.8% of patients without hydronephrosis. Together with these evidences, for those UTUC patients with pre-NU hydronephrosis, adjuvant CBCT seems to be more adequate than neoadjuvant setting.

The present study, however, has some limitations. First, our cohort was retrospective. Second, the degree of preoperative hydronephrosis was not documented; therefore, the correlation between hydronephrosis severity and RF decline could not be evaluated. Third, the long-term renal function follow-up data was lacking, however, it was beyond the scope of our study which focused on the short-term period of post-NU adjuvant therapy. Finally, the RF was determined using the eGFR estimation. However, more detailed nephrological information such as proteinuria, 24h urine creatinine clearance, renal scintigraphy or magnetic resonance urography was not available in the registry database.

## Conclusions

Our study confirmed that preoperative ipsilateral hydronephrosis is an independent protective predictor not only for post-NU RF decline in CKD stage II and III but also for CKD progression and preservation of eligibility for CBCT or IO therapy. With cautious selection for those unfavorable prognostic non-metastatic UTUC patients with preoperative hydronephrosis, adjuvant therapy following definitive NU rather than neoadjuvant setting could be considered due to higher chance of preserving eligibility for adjuvant treatment.

## Data availability statement

The raw data supporting the conclusions of this article will be made available by the authors, without undue reservation.

## Ethics statement

The studies involving human participants were reviewed and approved by 108140-E. Written informed consent for participation was not required for this study in accordance with the national legislation and the institutional requirements.

## Author contributions

P-YC contributed to acquisition of data, analysis and interpretation of data, drafting of the manuscript and statistical analysis. C-YT contributed to conception and design, analysis and visualization of data, critical revision of the manuscript, statistical analysis and supervision. All authors collected the data.

## References

[1] Siegel RL Miller KD Jemal A. Cancer statistics, 2019. CA: Cancer J Clin (2019) 69(1):7–34. doi: 10.3322/caac.21551 30620402

[2] Visser O Adolfsson J Rossi S Verne J Gatta G Maffezzini M. Incidence and survival of rare urogenital cancers in Europe. Eur J cancer (2012) 48(4):456–64. doi: 10.1016/j.ejca.2011.10.031 22119351

[3] Seisen T Peyronnet B Dominguez-Escrig JL Bruins HM Yuan CY Babjuk M. Oncologic outcomes of kidney-sparing surgery versus radical nephroureterectomy for upper tract urothelial carcinoma: A systematic review by the EAU non-muscle invasive bladder cancer guidelines panel. Eur urol (2016) 70(6):1052–68. doi: 10.1016/j.eururo.2016.07.014 27477528

[4] Lane BR Smith AK Larson BT Gong MC Campbell SC Raghavan D. Chronic kidney disease after nephroureterectomy for upper tract urothelial carcinoma and implications for the administration of perioperative chemotherapy. Cancer (2010) 116(12):2967–73. doi: 10.1002/cncr.25043 20564402

[5] Outcomes KDIG Group CW. KDIGO 2012 clinical practice guideline for the evaluation and management of chronic kidney disease. Kidney Int (2013) 3(1):1–150. doi: 10.1038/kisup.2012.64 23989362

[6] Huang WC Levey AS Serio AM Snyder M Vickers AJ Raj GV. Chronic kidney disease after nephrectomy in patients with renal cortical tumours: A retrospective cohort study. Lancet Oncol (2006) 7(9):735–40. doi: 10.1016/S1470-2045(06)70803-8 PMC2239298 16945768

[7] Yafi FA Tanguay S Rendon R Jacobsen N Fairey A Izawa J. Adjuvant chemotherapy for upper-tract urothelial carcinoma treated with nephroureterectomy: Assessment of adequate renal function and influence on outcome. In: Urologic oncology. (2014) 32(1):e17-24. doi: 10.1016/j.urolonc.2012.11.014 23428535

[8] Bajorin DF Witjes JA Gschwend JE Schenker M Valderrama BP Tomita Y. Adjuvant nivolumab versus placebo in muscle-invasive urothelial carcinoma. New Engl J Med (2021) 384(22):2102–14. doi: 10.1056/NEJMoa2034442 PMC8215888 34077643

[9] Birtle AJ Chester JD Jones RJ Johnson M Hill M Bryan RT. Adjuvant chemotherapy in upper tract urothelial carcinoma (the POUT trial): A phase 3, open-label, randomised controlled trial. The Lancet. (2020) 395(10232), 1268–77. 10.1016/S0140-6736(20)30415-3 PMC7181180 32145825

[10] Galsky MD Hahn NM Rosenberg J Sonpavde G Hutson T Oh WK. Treatment of patients with metastatic urothelial cancer “unfit” for cisplatin-based chemotherapy. J Clin Oncol (2011) 29(17):2432–8. doi: 10.1200/JCO.2011.34.8433 21555688

[11] EEIG B-MSP. Opdivo summary of product characteristics (2015). European Union (Accessed 2021 Dec 30]).

[12] Hoshino K Kikuchi E Tanaka N Akita H Ito Y Miyajima A. Preoperative hydronephrosis: Independent predictor for changes in renal function following nephroureterectomy. Japanese J Clin Oncol (2012) 42(3):202–7. doi: 10.1093/jjco/hyr199 22246718

[13] Singla N Hutchinson R Haddad A Sagalowsky A Lotan Y Margulis V. Preoperative hydronephrosis is associated with less decline in renal function after radical nephroureterectomy for upper tract urothelial carcinoma. Can J urol (2016) 23(4):8334–41. 27544555

[14] Inker LA Eneanya ND Coresh J Tighiouart H Wang D Sang Y. New creatinine-and cystatin c–based equations to estimate GFR without race. New Engl J Med (2021) 385(19):1737–49. doi: 10.1056/NEJMoa2102953 PMC8822996 34554658

[15] Levey AS Stevens LA Schmid CH Zhang Y Castro AF III Feldman HI. A new equation to estimate glomerular filtration rate. Ann Internal Med (2009) 150(9):604–12. doi: 10.7326/0003-4819-150-9-200905050-00006 PMC2763564 19414839

[16] Levey AS Coresh J Greene T Stevens LA Zhang Y Hendriksen S. Using standardized serum creatinine values in the modification of diet in renal disease study equation for estimating glomerular filtration rate. Ann Internal Med (2006) 145(4):247–54. doi: 10.7326/0003-4819-145-4-200608150-00004 16908915

[17] Chen L-I Guh J-Y Wu K-D Chen Y-M Kuo M-C Hwang S-J. Modification of diet in renal disease (MDRD) study and CKD epidemiology collaboration (CKD-EPI) equations for Taiwanese adults. PloS One (2014) 9(6):e99645. doi: 10.1371/journal.pone.0099645 24927124 PMC4057229

[18] Chang P-Y Chien L-N Lin Y-F Wu M-S Chiu W-T Chiou H-Y. Risk factors of gender for renal progression in patients with early chronic kidney disease. Medicine (2016) 95(30):e4203. doi: 10.1097/MD.0000000000004203 27472690 PMC5265827

[19] Martini A Cumarasamy S Beksac AT Abaza R Eun DD Bhandari A. A nomogram to predict significant estimated glomerular filtration rate reduction after robotic partial nephrectomy. Eur Urol (2018) 74(6):833–9. doi: 10.1016/j.eururo.2018.08.037 30224195

[20] Anari F O’Neill J Choi W Chen DY Haseebuddin M Kutikov A. Neoadjuvant dose-dense gemcitabine and cisplatin in muscle-invasive bladder cancer: Results of a phase 2 trial. Eur Urol Oncol (2018) 1(1):54–60. doi: 10.1016/j.euo.2018.02.007 30420974 PMC6226048

[21] Dicker S Shirley D. Compensatory hypertrophy of the contralateral kidney after unilateral ureteral ligation. J Physiol (1972) 220(1):199. doi: 10.1113/jphysiol.1972.sp009701 5059234 PMC1331696

[22] Jang SJ Choi BS Choi SH. Evaluation of renal function in obstructed ureter model using 99mTc-DMSA. vivo (2020) 34(5):2431–5. doi: 10.21873/invivo.12057 PMC7652498 32871769

[23] Kaag MG O'Malley RL O'Malley P Godoy G Chen M Smaldone MC. Changes in renal function following nephroureterectomy may affect the use of perioperative chemotherapy. Eur urol (2010) 58(4):581–7. doi: 10.1016/j.eururo.2010.06.029 PMC3677959 20619530

[24] Singla N Hutchinson R Menegaz C Haddad AQ Jiang L Sagalowsky AI. Comparing changes in renal function after radical surgery for upper tract urothelial carcinoma and renal cell carcinoma. Urology (2016) 96:44–53. doi: 10.1016/j.urology.2016.07.015 27443467

[25] Seisen T Krasnow RE Bellmunt J Rouprêt M Leow JJ Lipsitz SR. Effectiveness of adjuvant chemotherapy after radical nephroureterectomy for locally advanced and/or positive regional lymph node upper tract urothelial carcinoma. J Clin Oncol (2017) 35(8):852–60. doi: 10.1200/JCO.2016.69.4141 28045620

[26] Kaag M Trost L Thompson RH Favaretto R Elliott V Shariat SF. Preoperative predictors of renal function decline after radical nephroureterectomy for upper tract urothelial carcinoma. BJU Int (2014) 114(5):674–9. doi: 10.1111/bju.12597 24314050

[27] Chung JS Son NH Byun SS Lee SE Hong SK Jeong CW. Trends in renal function after radical nephrectomy: A multicentre analysis. BJU Int (2014) 113(3):408–15. doi: 10.1111/bju.12277 23937424

[28] Zabor EC Furberg H Mashni J Lee B Jaimes EA Russo P. Factors associated with recovery of renal function following radical nephrectomy for kidney neoplasms. Clin J Am Soc Nephrol (2016) 11(1):101–7. doi: 10.2215/CJN.04070415 PMC4702228 26500248

[29] Leow JJ Martin-Doyle W Fay AP Choueiri TK Chang SL Bellmunt J. A systematic review and meta-analysis of adjuvant and neoadjuvant chemotherapy for upper tract urothelial carcinoma. Eur urol (2014) 66(3):529–41. doi: 10.1016/j.eururo.2014.03.003 24680361

[30] Quhal F Mori K Motlagh RS Laukhtina E Pradere B Rouprêt M. Efficacy of neoadjuvant and adjuvant chemotherapy for localized and locally advanced upper tract urothelial carcinoma: A systematic review and meta-analysis. Int J Clin Oncol (2020) 25(6):1037–54. doi: 10.1007/s10147-020-01650-9 32206939

[31] Leow JJ Chong YL Chang SL Valderrama BP Powles T Bellmunt J. Neoadjuvant and adjuvant chemotherapy for upper tract urothelial carcinoma: A 2020 systematic review and meta-analysis, and future perspectives on systemic therapy. Eur Urol (2021) 79(5):635–54. doi: 10.1016/j.eururo.2020.07.003 32798146

[32] Margolin EJ Matulay JT Li G Meng X Chao B Vijay V. Discordance between ureteroscopic biopsy and final pathology for upper tract urothelial carcinoma. J Urol (2018) 199(6):1440–5. doi: 10.1016/j.juro.2018.02.002 29427584

[33] Lucca I Leow JJ Shariat SF Chang SL. Diagnosis and management of upper tract urothelial carcinoma. Hematol/Oncol Clinics. (2015) 29(2):271–88. doi: 10.1016/j.hoc.2014.10.003 25836934

[34] Gayed BA Thoreson GR Margulis V. The role of systemic chemotherapy in management of upper tract urothelial cancer. Curr Urol Rep (2013) 14(2):94–101. doi: 10.1007/s11934-013-0307-z 23344684

[35] Rouprêt M Colin P Yates DR. A new proposal to risk stratify urothelial carcinomas of the upper urinary tract (UTUCs) in a predefinitive treatment setting: Low-risk versus high-risk UTUCs. Eur urol (2013) 66(2):181–3. doi: 10.1016/j.eururo.2013.12.007 24361259

[36] Seisen T Colin P Roupret M. Risk-adapted strategy for the kidney-sparing management of upper tract tumours. Nat Rev Urol (2015) 12(3):155–66. doi: 10.1038/nrurol.2015.24 25708579

[37] Chen I-HA Chang C-H Huang C-P Wu W-J Li C-C Chen C-H. Factors predicting oncological outcomes of radical nephroureterectomy for upper tract urothelial carcinoma in Taiwan. Front Oncol (2021) 11. doi: 10.3389/fonc.2021.766576 PMC8793058 35096575

[38] Lee JN Kwon SY Choi GS Kim HT Kim TH Kwon TG. Impact of surgical wait time on oncologic outcomes in upper urinary tract urothelial carcinoma. J Surg Oncol (2014) 110(4):468–75. doi: 10.1002/jso.23589 25059848

