# Supplementary material for: Preoperative hydronephrosis is an independent protective factor of renal function decline after nephroureterectomy for upper tract urothelial carcinoma
Source: Front Oncol. 2023 Feb 24;13:944321. doi: 10.3389/fonc.2023.944321 (PMC9998910; doi:10.3389/fonc.2023.944321)
Supplement: Supplementary file 1 [file DataSheet_1.pdf]

Supplementary Documents

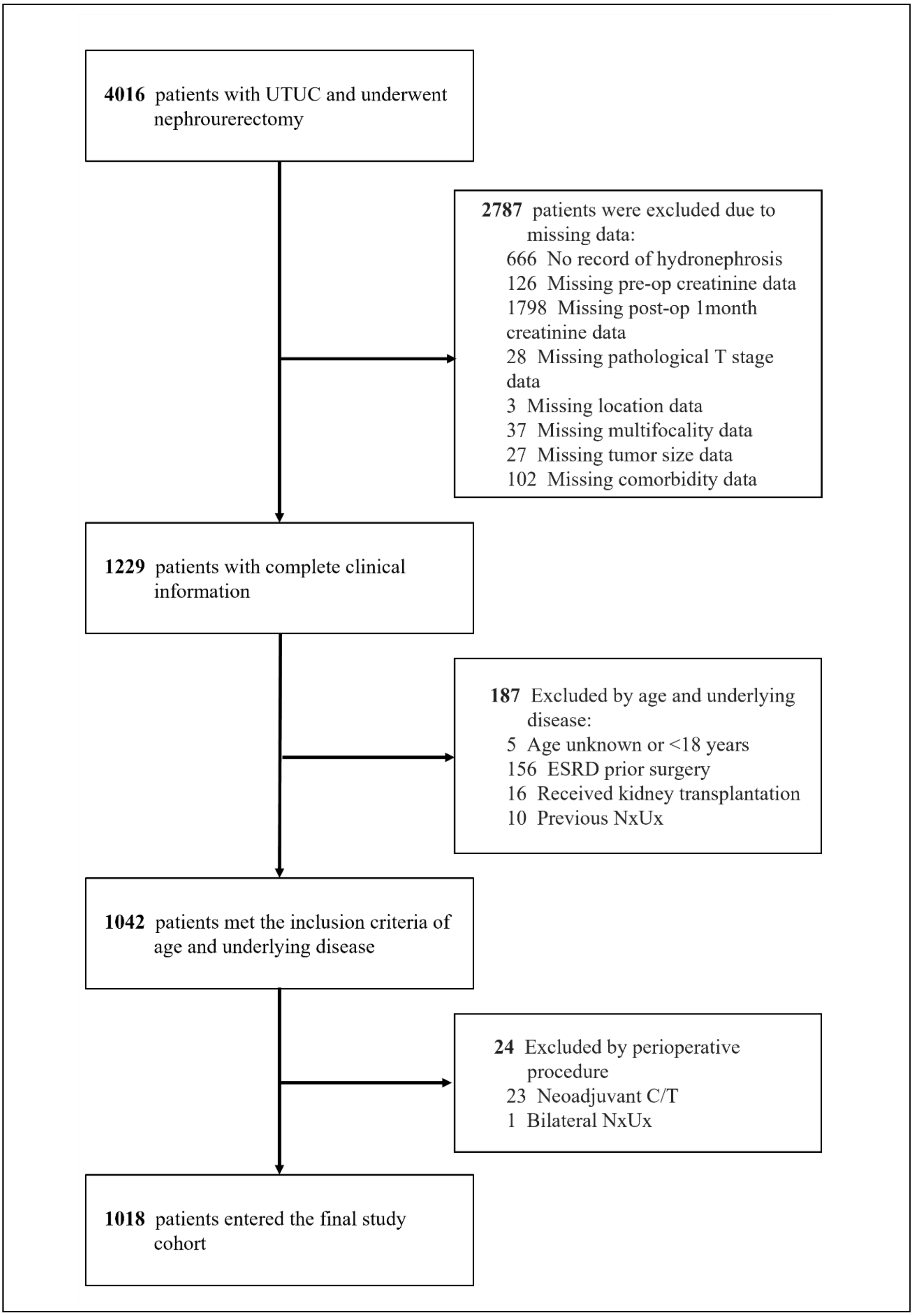

Figure S1. Flowchart of study process

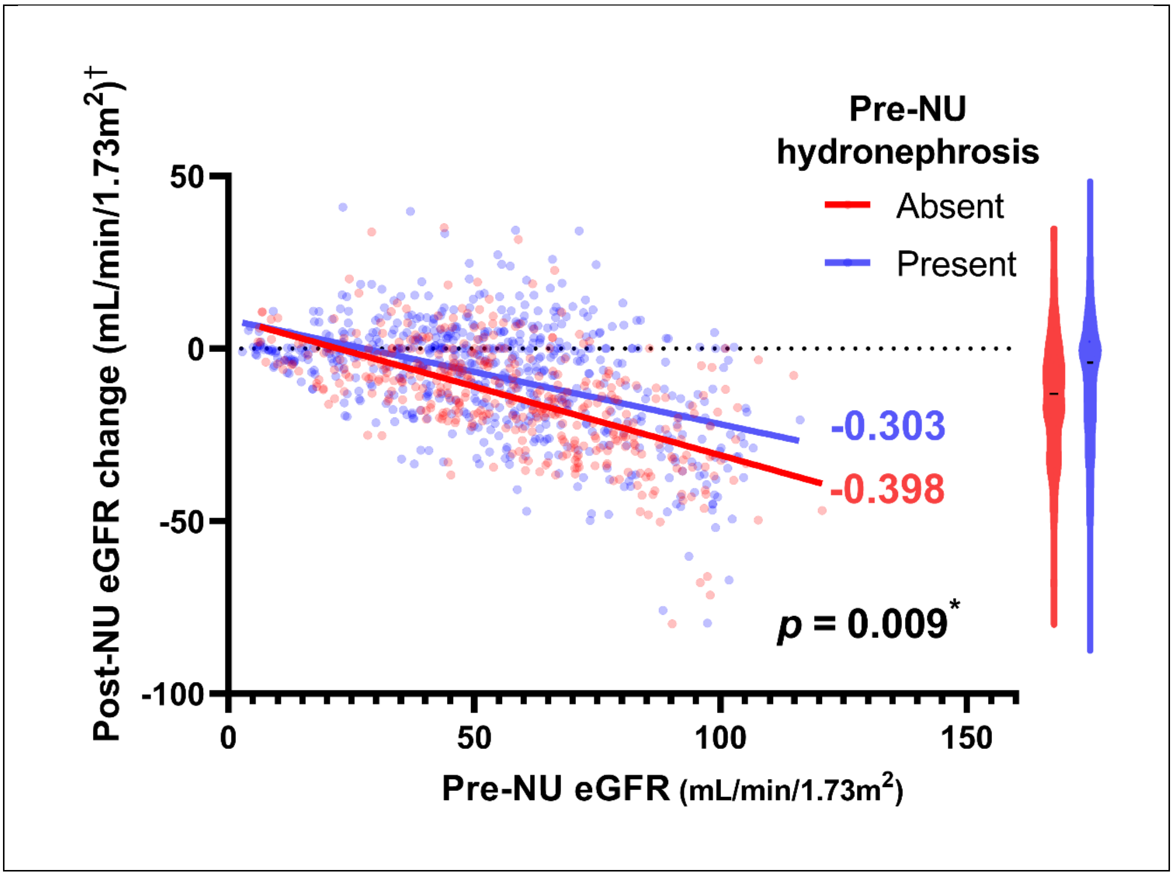

Figure S2. Scatter plot and regression line of post-NU ΔeGFR versus pre-NU eGFR.

†Post-NU ΔeGFR = (1 month post-NU eGFR) – (pre-NU eGFR)

* Statistically significant

Variable¶	Univariable analysis			Multivariable analysis		
	OR	(95% CI)	*p* value	OR	(95% CI)	*p* value
Hydronephrosis	0.51	(0.39 – 0.66)	<0.001*	0.50	(0.39 – 0.65)	<0.001*
Age	1.00	(0.99 – 1.01)	0.753	-	-	-
Women	0.94	(0.73 – 1.21)	0.653	-	-	-
Hypertension	0.99	(0.77 – 1.28)	0.946	-	-	-
Diabetes	1.10	(0.84 – 1.45)	0.472	-	-	-
Coronary artery disease	1.15	(0.74 – 1.78)	0.544	-	-	-
Left side	0.90	(0.70 – 1.16)	0.428	-	-	-
Ureter involvement	0.53	(0.41 – 0.68)	<0.001*	-	-	-
Tumor focality	0.74	(0.57 – 0.96)	0.023*	-	-	-
Tumor size ≥ 2 cm	0.71	(0.53 – 0.95)	0.020*	-	-	-
Pathologic T stage ≥ pT3	0.64	(0.50 – 0.82)	0.001*	0.63	(0.48 – 0.81)	<0.001*
Post-NU shock	2.69	(0.78 – 9.25)	0.116	3.61	(1.03 – 12.74)	0.046

Table S1. Logistic regression of post-NU CKD progression† (incremental progression to a higher CKD stage) (n = 1018)

OR: Odds ratio

† CKD progression = CKD progresses to higher stage after NU

¶ Dichotomous variable compared to the opposite character, eg: hydronephrosis vs no hydronephrosis (reference)

* Statistically significant

Pre-NU CKD stage		n	ΔeGFR[†]			Pre-NU hydronephrosis					Pre-NU hydronephrosis in MLR[¶]	
						Absent		Present		*p value*		
			Mean	%[‡]	SD	Mean	SD	Mean	SD		Coefficient (B)	*p value*
I	eGFR ≥ 90	100	-30.26	-31.1%	17.44	-32.04	18.37	-29.02	16.81	0.406	2.38	0.513
II	60 ≤ eGFR < 90	316	-15.52	-21.0%	16.20	-21.08	12.90	-11.15	17.19	< 0.001*	9.27	< 0.001*
III	30 ≤ eGFR < 60	431	-5.15	-11.0%	12.69	-7.13	11.67	-3.86	13.17	0.009*	3.79	0.002*
IV	15 ≤ eGFR < 30	111	-1.31	-6.3%	9.921	-3.49	10.59	-0.23	9.46	0.103	3.70	0.054
V	eGFR < 15	60	-0.41	4.0%	4.54	1.18	6.20	-0.98	3.70	0.205	-3.14	0.023*

Table S2. Post-NU eGFR change[†] in two groups stratified by pre-NU CKD stage (n = 1018)

SD: Standard deviation

† ΔeGFR = Post-NU eGFR change = (1 month post-NU eGFR) – (pre-NU eGFR)

‡ Post-NU eGFR change (%) = $\frac{(1\ month\ post-NU\ eGFR) - (pre-NU\ eGFR)}{(pre-NU\ eGFR)}$

¶ Multiple linear regression adjusted for age, hypertension, tumor focality, pathological p stage, pre-NU eGFR

* Statistically significant
